# Characterization, Expression Profile, and Promoter Analysis of the *Rhodeus uyekii* Vitellogenin Ao1 Gene

**DOI:** 10.3390/ijms151018804

**Published:** 2014-10-17

**Authors:** Hee Jeong Kong, Ju Lan Kim, Ji Young Moon, Woo-Jin Kim, Hyung Soo Kim, Jung Youn Park, Hyun Kook Cho, Cheul Min An

**Affiliations:** 1 Biotechnology Research Division, National Fisheries Research and Development Institute, Busan 619-705, Korea; E-Mails: heejkong@korea.kr (H.J.K.); tks1010@hanmail.net (J.L.K.); moonjy@pknu.ac.kr (J.Y.M.); wj2464@korea.kr (W.-J.K.); ecomarine@korea.kr (H.S.K.); genome@korea.kr (J.Y.P.); ancm@korea.kr (C.M.A.); 2 Department of Molecular Biology, Pusan National University, Busan 609-735, Korea

**Keywords:** 17β-Estradiol, 17α-Ethinylestradiol, Korean rose bitterling, promoter assay, *Rhodeus uyekii*, vitellogenin

## Abstract

The fish Vitellogenin (Vg) gene has been applied as a biomarker for exposure to estrogenic compounds in the aquatic environment. In this study, we cloned and characterized Vg cDNA from the Korean rose bitterling *Rhodeus uyekii* (Ru-Vg). The Ru-Vg cDNA encodes a 1424-amino-acid polypeptide that belongs to the VgAo1 family and contains a putative signal peptide, lipovitellin I, phosvitin, and lipovitellin II, but does not contain the vWFD domain or the *C*-terminal peptide. The deduced Ru-Vg protein has high amino acid identity (73.97%–32.17%) with fish Vg proteins. Pairwise alignment and phylogenetic analysis revealed that Ru-Vg is most closely related to *Acheilognathus yamatsutae* Vg. Ru-Vg transcripts were detected using quantitative polymerase chain reaction in all tissues tested, with the highest level of expression observed in the ovary. Ru-Vg mRNA was upregulated in *R. uyekii* hepatopancreas cells in response to treatment with 17β-estradiol (E2) or 17α-ethinylestradiol (EE2). Luciferase reporter expression, driven by the 5'-regulatory region of the Ru-Vg gene spanning from −1020 bp to the start codon was induced by the estrogen receptor and was synergistically activated by treatment with E2 or EE2. These results suggest that *R. uyekii* and the Ru-Vg gene may be useful as biomarkers for exposure to E2 or EE2.

## 1. Introduction

Vitellogenin (Vg) is a precursor protein of vitellin (Vn; a major egg yolk protein that plays a major role in vitellogenesis), an important reproductive process in oviparous animals. Vg is a calcium-binding glycolipophosphoprotein that consists of (from *N*- to *C*-terminus) a signal peptide, lipovitellin-I (heavy chain), phosvitin, lipovitellin-II (light chain), Von Willebrand factor type D (vWFD), and a *C*-terminal peptide [1]. Vg is generally synthesized in the liver of oviparous females in response to 17β-estradiol (E2) produced by the developing ovary [2]. It is then transported via the bloodstream to the follicular layer, where it is taken up by developing oocytes via receptor-mediated endocytosis [3,4]. After endocytosis, Vg is proteolytically cleaved into several domains; the major domains of Vg are lipovitellin and phosvitin. Lipovitellin serves as a source of amino acids and lipids [5], and phosvitin, which contains highly phosphorylated polyserine domains, acts as a binding protein for several metals in the yolk [6] and is implicated in receptor binding during endocytosis in insects [7]. Although the functions of the vWFD and *C*-terminal peptide are still unknown with respect to embryonic nutrition, the CGXC motif of the vWFD and the polycysteine residues of the *C*-terminal peptide have been proposed to participate in the process of vitellogenin polypeptide folding [1,8,9].

Although males also possess the Vg gene, Vg mRNA is undetectable in males; under normal conditions, Vg gene expression is female-specific. Therefore, the Vg gene can be applied as a sensitive indicator of gender and ovarian development in female fish [10,11,12]. However, the Vg gene can be induced in males by artificial estrogens or estrogen-like compounds [13]. Sustaining levels of plasma Vg in males exposed to estrogenic compounds is due to the absence of a mechanism to clear plasma Vg, a task performed by the oocytes in females [14]. For this reason, the fish Vg gene has been extensively applied as a biomarker for exposure to estrogenic compounds in the aquatic environment [15].

In addition to estrogen as a stimulator of Vg gene expression, farnesoic acid (FA) and 20-hydroxyecdysone (20E) also stimulate Vg gene expression in lobsters [16]. Conversely, vitellogenesis-inhibiting hormone (VIH) is a peptide hormone that suppresses Vg gene expression in Pacific white shrimp [17]. The estrogen receptor (ER) is a transcription factors that is essential for estrogen-induced Vg expression. Once activated by estrogen, ER translocates into the nucleus and binds to the estrogen-response element (ERE) in the promoter region of genes including Vg, thereby regulating expression [18,19]. Hepatocyte nuclear factor 3 (HNF3) and ER activate Vg gene expression synergistically in African clawed frog oocytes [20]. In addition, GATA, vitellogenin binding protein (VBP), and ER have a synergistic effect on Vg gene expression in the blue tilapia [21]. In addition to the activators for Vg gene expression, cAMP response element binding protein (CREB), and heat shock cognate 70 (HSC70) repress Vg gene expression in the mosquito fat body and in the shrimp ovary and hepatopancreas, respectively [22,23].

Bitterlings are of the *Acheilognathinae* subfamily, and are distributed across temperate regions of Europe and Asia, including China, Japan, and Korea [24]. Korean bitterlings are classified into two genera and 14 species, including nine endemic species [25]. The Korean rose bitterling *Rhodeus uyekii* is a fish endemic to Korea found in rivers that empty into the Western and Southern Sea of Korea. Recently, genetic studies on the Korean rose bitterling have been initiated; Kim *et al.* reported the complete mitochondrial genome sequence of *R. uyekii* [26]. To date, the *R. uyekii* Vg gene has not been characterized. In this paper, we report the isolation of an *R. uyekii* Vg (Ru-Vg) cDNA and describe the tissue-specific expression of the Ru-Vg gene. We also characterize the promoter region of Ru-Vg gene, and identify the ERE. Finally, we demonstrate the activation of the Ru-Vg promoter by estrogen or estrogenic compounds using a luciferase assay system.

## 2. Results and Discussion

### 2.1. Molecular Characterization of the R. uyekii Vitellogenin Gene

**Figure 1:**
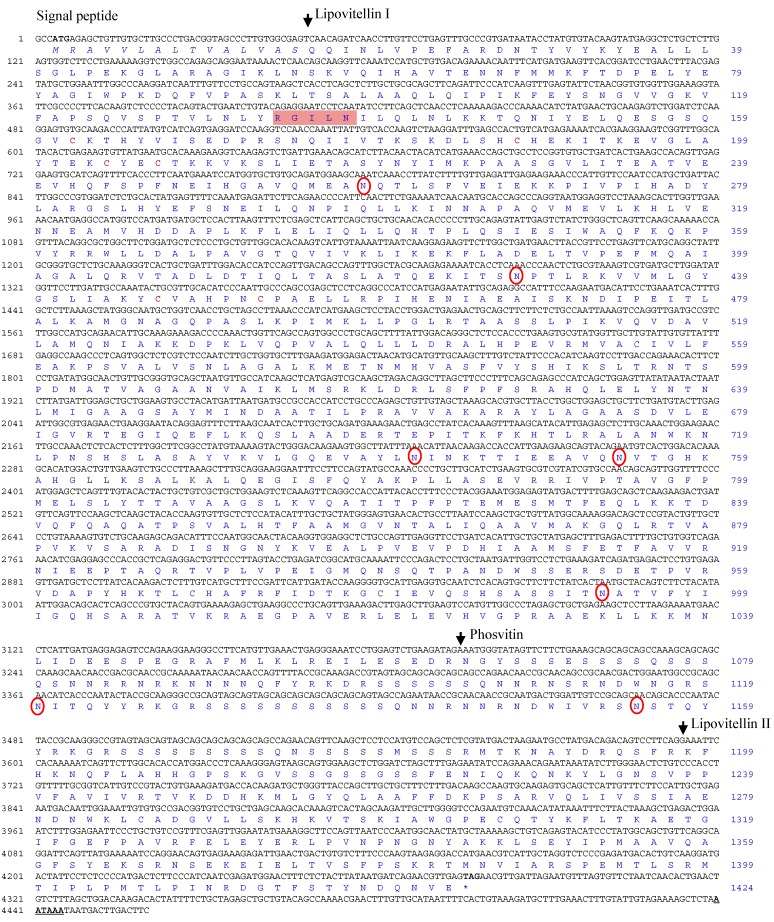
Nucleotide and deduced amino acid sequences of Ru-Vg cDNA. Nucleotides (black) and amino acids (blue) are numbered on the left and right sides of each line, respectively. Start (ATG) and stop (TAA) codons are indicated in bold. Putative signal peptide sequences are shown in italics. The boundaries of the three distinct domains, lipovitellin I, phosvitin, and lipovitellin II, are indicated by arrows. The disulfide bond is indicated in bold and brown, and the *N*-glycosylation sites are denoted by red rings. The conserved motif (RGILN) of Ru-Vg is in a pink box. Polyadenylation signals (AATAAA) are bold and underlined.

The Ru-Vg cDNA sequence was isolated from the expressed sequence tag (EST) analysis of the Korean rose bittering *R. uyekii* cDNA library (data not shown). The Ru-Vg cDNA (accession number KM111547) was 4460 bp in length and contained an open reading frame (ORF) of 4272 bp, encoding a protein of 1424 amino acids (Figure 1). The theoretical isoelectric point (pI) and molecular weight (MW) of the deduced Ru-VG protein were calculated as 9.25 and 157.4 kDa, respectively. A total of seven *N*-glycosylation sites (N259, 428, 742, 754, 993, 1120, 1155) and three disulfide bonds (C162–C188, C204–C207, C447–C453) were predicted in the deduced Ru-Vg protein sequence. The deduced Ru-Vg protein contained a putative signal peptide (M1–S15) with a cleavage site between S15 and Q16, lipovitellin I (Q16–R1064; including the conserved RGILN motif), phosvitin (N1065–R1197), and lipovitellin II (K1198–F1424; lacking the *C*-terminal half including the conserved TCGL/ICG motif). The deduced protein did not, however, contain the Von Willebrand factor type D (vWFD) or *C*-terminal peptide domains.

Three types of Vg transcripts were detected in fish, including mosquitofish, mummichog, red seabream, white perch, and gray mullet [27,28,29,30,31]. Two of the types were designated as complete VgA (or Vg-I) and VgB (or Vg-II), containing all of the yolk protein domains (lipovitellin, phosvitin, vWFD, and *C*-terminal peptide), and later renamed as VgAa and VgAb by Finn and Kristoffersen [32]. The third type was designated incomplete VgC, lacking phosvitin and the *C*-terminal peptide domain (Pv-less type) [33]. The deduced Ru-Vg lacks the vWFD and *C*-terminal peptide domains, similar to the Vgs of the common carp, fathead minnow, zebrafish, Chinese rare minnow, and white cloud mountain minnow [34,35,36]. According to the nomenclature of Finn and Kristoffersen [32], Vg lacking the vWFD and *C*-terminal peptide domains is classified as VgAo1; therefore, Ru-Vg is the VgAo1 type. Vg lacking lipovitellin has not yet been reported, suggesting that the lipovitellin domain plays a major role in Vg function. The vWFD domain of Vg has been demonstrated to be necessary for normal multimerization and optimal secretion [37], which suggests that the Ru-Vg protein may function as a monomer and that the vWFD domain may not be essential for Ru-Vg activity.

### 2.2. Pairwise Alignment and Phylogenetic Analysis of Vg Proteins

**Figure 2:**
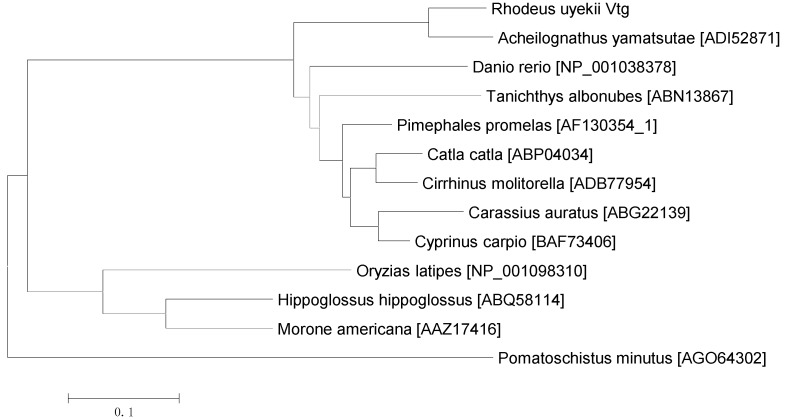
Phylogenetic analysis of Vg family members. An unrooted phylogenetic tree was constructed using the neighbor-joining method. The sequences were extracted from GenBank. The accession numbers are indicated in the figure.

Pairwise sequence alignment shows that among other known Vgs, Ru-Vg possesses the highest amino acid identity with *Acheilognathus yamatsutae* Vg (73.97% identity), followed by *Cyprinus carpio* Vg (70.38%), *Pimephales promelas* Vg (70.17%), *Cirrhinus molitorella* Vg (69.09%), *Catla catla* Vg (68.57%), *Carassius auratus* Vg (66.48%), *Tanichthys albonubes* Vg (64.06%), *Danio rerio* Vg (54.85%), *Morone americana* Vg (40.20%), *Hippoglossus hippoglossus* Vg (40.05%), *Oryzias latipes* Vg (38.04%), and *Pomatoschistus minutes* Vg (32.17%) (Table 1). To determine the evolutionary relationship between Ru-Vg and other Vg proteins, a phylogenetic tree of 13 fish Vg protein sequences was constructed using Mega 5.0 software and the neighbor-joining method. As shown in Figure 2, Ru-Vg is most closely related to *A. yamatsutae* Vg, with which it forms a clade.

**Table 1:** Pairwise sequence alignment of Ru-Vg with selected Vg amino acid sequences.

Species	Accession Number	Identity (%)	Amino Acids
*Acheilognathus yamatsutae*	ADI52871	73.97	1550
*Cyprinus carpio*	BAF73406	70.38	1353
*Pimephales promelas*	AF130354_1	70.17	1340
*Cirrhinus molitorella*	ADB77954	69.09	1342
*Catla catla*	ABP04034	68.57	1339
*Carassius auratus*	ABG22139	66.48	1348
*Tanichthys albonubes*	ABN13867	64.06	1326
*Danio rerio*	NP_001038378	54.58	1631
*Morone americana*	AAZ17416	40.20	1682
*Hippoglossus hippoglossus*	ABQ58114	40.05	1647
*Oryzias latipes*	NP_001098310	38.04	1725
*Pomatoschistus minutes*	AGO64302	32.17	1646

### 2.3. Expression of Ru-Vg mRNA in Tissues of R. uyekii

**Figure 3:**
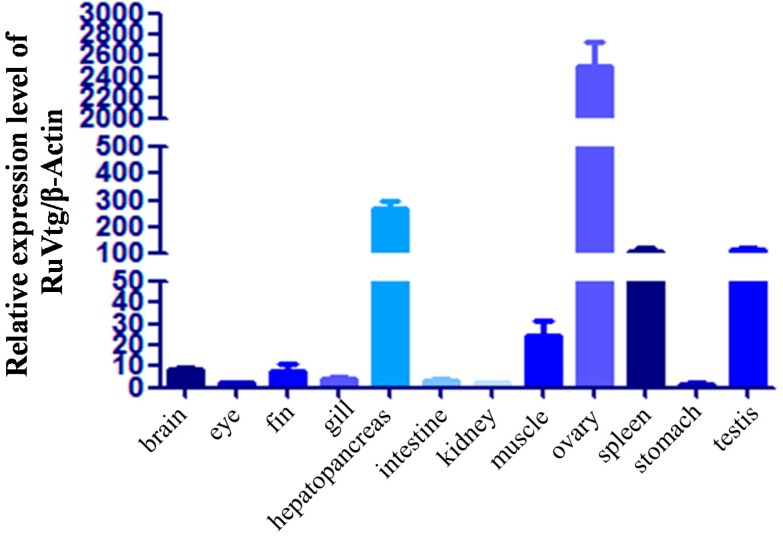
Real-time PCR analysis of Ru-Vg in tissues of *Rhodeus uyekii*. Quantitative real-time PCR was performed on equal amounts of total RNA isolated from Korean rose bitterling tissues. Transcript levels were quantified relative to β-actin. To determine tissue-specific expression levels, the expression level in each tissue was compared to that in the stomach.

To investigate the tissue distribution of Ru-Vg transcripts, quantitative real-time polymerase chain reaction (PCR) was performed using various tissues obtained from normally conditioned *R. uyekii* (Figure 3). The expression levels of Ru-Vg transcripts were estimated after normalization to β-actin as an internal reference gene. Ru-Vg transcripts were expressed in all tissues tested, but the relative expression levels of the transcripts were very low in all tissues except the hepatopancreas, ovary, spleen, and testis. The relative expression levels of Ru-Vg transcripts were 269-, 2508-, 109-, and 115-fold greater in the hepatopancreas, ovary, spleen, and testis, respectively, compared to the stomach. Vg is predominantly expressed in the liver or the hepatopancreas of vertebrates, the fat bodies of insects, and the intestine of nematodes [38,39,40]. In this study, Ru-Vg was predominantly expressed in the ovary. In crustaceans, some earlier studies demonstrated that Vg expression is restricted to the ovary, but Vg was also shown to be expressed exclusively in the hepatopancreas of some crustaceans. Other studies have reported that Vg is expressed in both the ovary and hepatopancreas in several crustaceans [17,41]. In some fish, Vg is expressed in the heart, brain, and liver of the zebrafish and in the brain and heart of the Chinese rare minnow [34,35]. In addition, similar to Ru-Vg, the Vg of the white cloud mountain minnow was expressed at a higher level in the ovary than in the liver [36]. Ru-Vg and all of the Vg genes mentioned above are AtgAo1 type [34,35,36]. In studies on the zebrafish and Chinese rare minnow, the researchers did not investigate Vg expression in the ovary. Therefore, we tentatively conclude that VgAo1 may be predominantly expressed in the ovary.

Some researchers have reported that the Vg gene isolated from the ovary is different from the Vg gene isolated from the hepatopancreas [1]. Thus, some fish have three Vg types [27,28,29,30,31]. These findings suggest that Vg genes of other types, which are predominantly expressed in the hepatopancreas, may exist in *R. uyekii*, and that Vg subtypes may perform tissue-specific roles.

### 2.4. Regulation of Ru-Vg mRNA by 17β-Estradiol (E2) or 17α-Ethinylestradiol (EE2) in R. uyekii Hepatopancreas Cells

**Figure 4:**
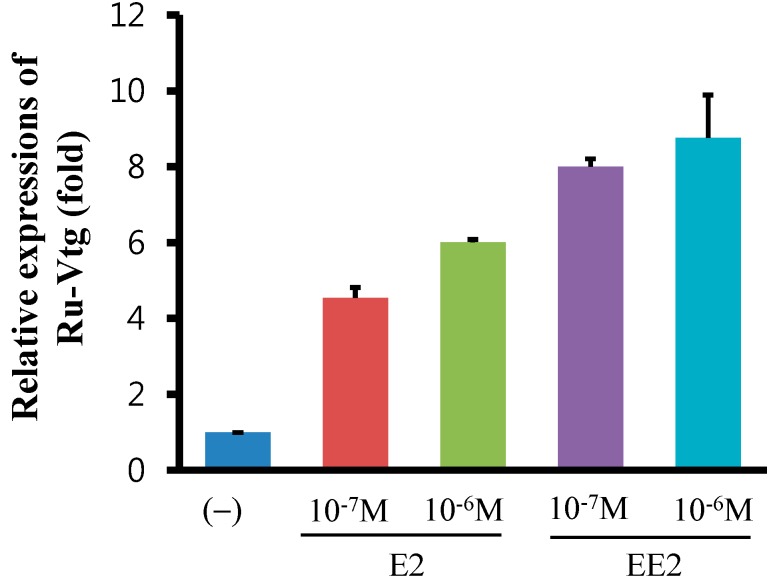
17β-Estradiol (E2)- and 17α-ethinylestradiol (EE2)-induced Ru-Vg transcription. Quantitative reverse transcription (RT)-PCR was performed on equal amounts of total RNA isolated from Korean rose bitterling hepatopancreas. Transcript levels were quantified relative to β-actin. Expression levels were calculated relative to the level of Ru-Vg in an unstimulated sample, which was arbitrarily defined as 1. PCR reactions were performed at least in triplicate.

To examine the effect of E2, the major endogenous estrogen in humans or EE2, a synthetic estrogen, on Ru-Vg gene expression, we isolated and cultured primary hepatopancreas cells from *R. uyekii*. The cells were exposed to E2 or EE2 and then processed for quantitative real-time PCR. As shown in Figure 4, Ru-Vg mRNA levels are induced approximately 4.53- and 6.0-fold or 8.0- and 8.87-fold in the primary hepatopancrease cells, by E2 and EE2 treatments, respectively. However, the effects of E2 or EE2 on Ru-Vg gene expression are significantly lower compared to those seen in other fish. In fish such as the slender bitterling, mud carp, zebrafish, and rainbow trout, the levels of hepatocyte Vg mRNA or plasma Vg were induced on a logarithmic scale by E2 or EE2 treatments [42,43,44,45]. In contrast, VgAo1 mRNA levels in the hepatocytes of zebrafish and white cloud mountain minnow were only induced approximately 1.5- and 10-fold by E2 treatment [34,36]. These results suggest that VgAo1 types in hepatocytes may be induced by E2 or EE2 treatments to a lesser extent than other Vg types. Several possible reasons exist for this diminished effect of E2 and EE2 on the expression of Ru-Vg or other VgAo1 genes: hepatocyte ER may have a low sensitivity for E2 or EE2, ER may be expressed at a low level in hepatocytes, and because the promoter region of the VgAo1 gene is packaged in hepatocytes, it may be difficult for ER to bind to the promoter.

### 2.5. Sequence Analysis of the 5'-Flanking Regions of the Ru-Vg Gene

**Figure 5:**
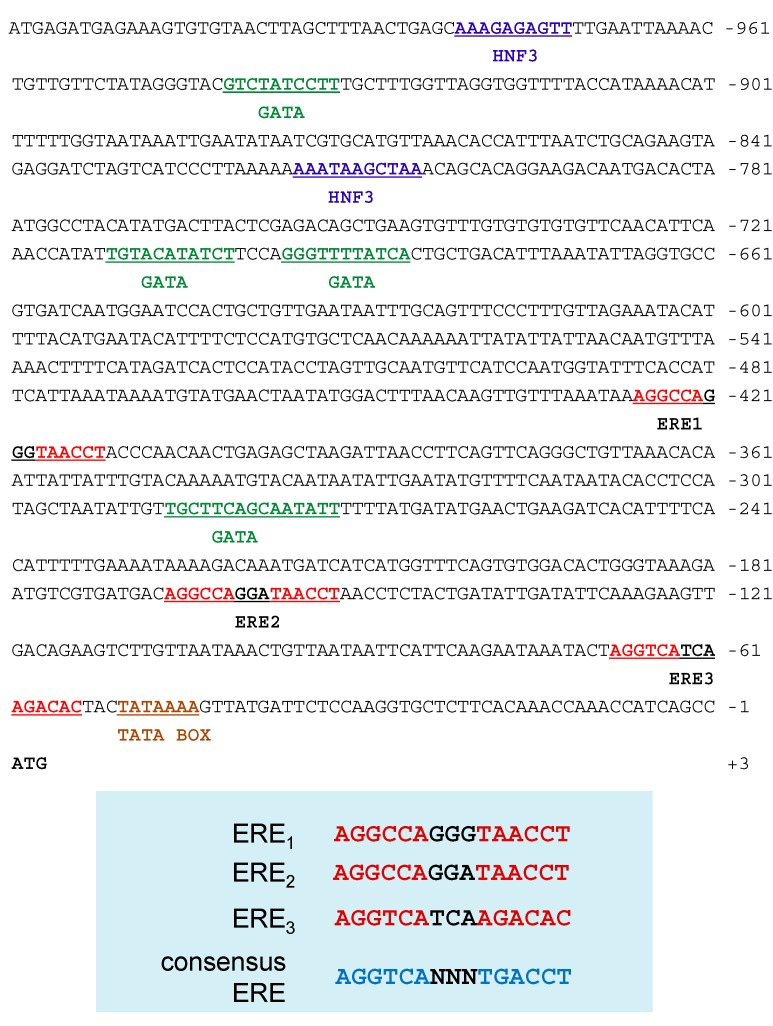
Sequence analysis of the 5'-flanking regions of the Ru-Vg gene. The locations of potential binding sites of HNF3 (blue), GATA (green), ERE (red), and TATA box (brown) are bold and underlined. HNF3 and GATA or ERE was found in threshold scores (default: 85.0 or 75.0), respectively. The A of the Ru-Vg start codon (ATG) is at the +1 position.

To investigate the mechanism underlying the regulation of Ru-Vg gene expression, we screened a *R. uyekii* fosmid library using primers based on the Ru-Vg cDNA sequence. We isolated the genomic clone #6-D07 containing the Ru-Vg gene covering from −1020 bp upstream from the putative transcription start site to the end of Ru-Vg cDNA sequence (accession number KM111548). We then analyzed the sequence using the TFSEARCH Web site and identified several putative transcription factor response elements (Figure 5). Three potential copies of an imperfect ERE were identified at position −427 to −413 bp (AGGCCAGGGTAACCT), −167 to −153 bp (AGGCCAGGATAACCT), and −69 to −55 bp (AGGTCATCAAGACAC) from the transcription start codon. The consensus ERE sequence is AGGTCAnnnTGACCT, but this is rarely found in estrogen-regulated genes [45]. Rather, a multitude of imperfect palindromic-like ERE sequences has been identified as functional EREs [45]. ER activated by the hormone estrogen (17β-estradiol) can regulate Vg gene expression through binding to the ERE. Predicted sites for liver-enriched transcription factor HNF3 (−982 to −973 bp and −816 to −806 bp) and GATA (−942 to −933 bp, −712 to −702 bp, −697 to −687 bp, and −287 to −273 bp) were also identified. HNF3 is expressed predominantly in the liver and plays important roles in the organization of chromatin structure and in the regulation of metabolism in metabolic tissues, such as the pancreas and liver [46]. GATA synergistically activated ER-mediated Vg transcription in the blue tilapia and chicken [21,47]. These findings suggest that regulation of the expression of Ru-Vg is complex, as in other orthologs, and is influenced by ER, HNF3, and/or GATA.

### 2.6. Functional Analysis of the 5'-Flanking Regions of the Ru-Vg Gene

**Figure 6:**
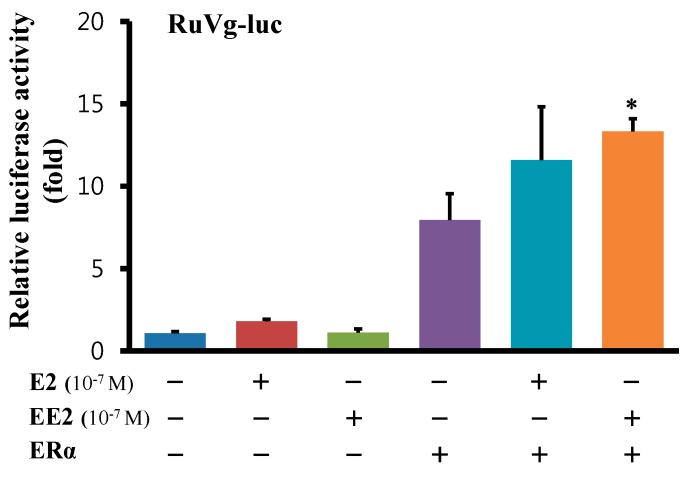
Functional analysis of the 5'-flanking regions of the Ru-Vg gene. Cells were seeded in 24-well culture plates and transfected with a Ru-Vg luciferase reporter vector and a β-galactosidase expression plasmid, together with an ERα expression plasmid. At 24 h after transfection, the cells were incubated in the presence or absence of E2 or EE2 for 16 h. At 48 h post-transfection, the cells were lysed and subjected to a luciferase reporter assay. Data are representative of two independent experiments and are presented as the mean ± SD (*n* = 3). * indicates *p* value < 0.05 with respect to the mock transfectants was deemed to indicate statistical significance.

To examine the activation of the 5'-flanking region of the Ru-Vg gene, we generated a luciferase reporter construct of the 5'-flanking region of Ru-Vg, and performed a luciferase assay to assess the effect of E2 and EE2 on transfected HEK293T cells. As shown Figure 6, the 5'-flanking region of Ru-Vg was activated by the expression of ERα, and this expression was synergistically increased by ERα together with 10^–7^ M E2 or 10^–7^ M EE2. These results suggest that Ru-Vg expression can be regulated by estrogen derivatives and indicate that *R. uyekii* and the Ru-Vg gene may have utility as biomarkers for exposure to endocrine-disrupting chemicals (EDCs), which can be harmful to human health by disrupting the endocrine, reproductive, and immune systems [42,48].

In addition to its function in vitellogenesis, Vg has some enzyme activity and has been shown to have defensive functions as an acute-phase immunity protein [43]. The presence of a HNF3-binding site in the Ru-Vg gene suggests that this gene may be regulated by HNF3, another major transcription factor expressed in the liver, in response to signals other than estrogen. This additional regulation pathway may be important for additional roles of Vg, such as those related to the immune response. Further studies are required to further elucidate the regulation of the Ru-Vg gene and the role of Ru-Vg in fish.

## 3. Experimental Section

### 3.1. Fish Maintenance and Tissue Samples

*R. uyekii* was collected from the Yangchun River, Uiryung-gun, Gyungnam, Republic of Korea. The fish were maintained at the National Fisheries Research and Development Institute (NFRDI) in Busan, Republic of Korea. The adults were maintained in 40-L glass aquaria at a density of approximately 20 fish per aquarium. The water was renewed weekly and the temperature in the rearing tanks was maintained at 20 ± 1 °C. The room was maintained on a 12 h/12 h light/dark cycle. Adults were fed TetraBits (Tetra, Melle, Germany) and frozen bloodworms (Advanced Hatchery Technology, Salt Lake City, UT, USA) twice a day. For RNA extraction, tissues were removed from three fish, immediately frozen in liquid nitrogen, kept separate, and stored at −80 °C before use.

### 3.2. Sequence Analyses and Phylogenetic Analyses

The Ru-Vg cDNA sequence was isolated from the EST analysis of the Korean rose bittering *R. uyekii* cDNA library (data not shown). EST clones were isolated from the *R. uyekii* cDNA library using a Plasmid Miniprep Kit (Qiagen, Seoul, Korea), and sequenced using T3 reverse primers (Promega, Madison, WI, USA) and an ABI3730xl automatic sequencer (Applied Biosystems, Foster City, CA, USA). Analyses of potential ORFs and comparisons of the Ru-Vg amino acid sequence (or nucleotide sequence) were performed using the ORF finder and BLAST programs [49]. The theoretical pI and the MW of the deduced Ru-Vg protein were computed on the ExPASy Website [50]. The signal sequence, putative *N*-glycosylation site, and putative disulfide bond were found using SignalP [51], the NetNGlyc1.0 Server [52], and the ScanProsite Server [53], respectively. Transcription factor binding sites were predicted using TFSEARCH [54]. A phylogenetic tree based on the deduced amino acid sequences was constructed using Mega 5 software [55] and the neighbor-joining algorithm. The reliability of branching was tested using bootstrap resampling with 1000 pseudo-replicates.

### 3.3. Quantitative Real-Time PCR

Total RNA was prepared from tissues using TRIzol reagent (Invitrogen, Carlsbad, CA, USA) according to the manufacturer’s instructions. The total RNA concentration was determined, and 1 μg was used for reverse transcription. First-strand cDNA was synthesized using an Advantage RT-for-PCR Kit (BD Sciences, San Jose, CA, USA). Quantitative real-time PCR was performed using Fast SYBR Green Master Mix (Applied Biosystems, Foster City, CA, USA) and the following forward and reverse primers: Ru-Vg, Ru-Vg-RT-F, 5'-GGA GTG TGC AAG ACC CAT TAT G-3'; Ru-Vg RT-R, 5'-AGT GGC TCA AAT CCT TAG ACT TGG T-3' and Ru-β-Actin (GenBank accession No. JQ279058), Ru-β-actin-F, 5'-GAT TCG CTG GAG ATG ATG CT-3'; Ru-β-actin-R, 5'-ATA CCG TGC TCA ATG GGG TA-3'. Following an initial 10-min Taq DNA polymerase activation step at 95 °C, real-time PCR was performed for 40 cycles using the following cycling conditions: 95 °C for 10 s, 60 °C for 15 s, followed by fluorescence reading in an SDS 7500 thermal cycler (Applied Biosystems). Ru-Vg transcript levels were quantified relative to β-actin transcript levels.

### 3.4. Isolation and Sequence Analysis of the Ru-Vg Gene

An *R. uyekii* fosmid library was screened to isolate the Ru-Vg gene using the fosmid pooling system, with PCR primers specific for the Ru-Vg cDNA. The primer sequences were RU-Vg_1F, 5'-CCT GCC AGT AAG CTC ACC TC-3'; RU-Vg _2R, 5'-GGA TTG GAA CAA TGG GTT TC-3'. PCR-based fosmid library screening was carried out as described previously [56]. The obtained Ru-Vg genomic clone #6-D07 was purified and used to determine the nucleotide sequence and genomic structure.

### 3.5. Cloning of the 5'-Flanking Region of the Ru-Vg Gene

To investigate the functional activity of the 5'-flanking region of the Ru-Vg gene, the DNA fragments (bp −1020 to +3 in the Ru-Vg gene) were generated by PCR using Vent DNA polymerase (New England BioLabs, Beverly, MA, USA). The primers were designed so that the amplified DNA would contain BglII and HindIII restriction sites at the 5' and 3' ends, respectively. The primer sequences were as follows: Ru-Vg Pro1023-F, 5'-CGG AGA TCT ATG AGA TGA GAA AGT-3'; Ru-Vg Pro1023-R, 5'-GAA AAG CTT CAT GGC TGA TGG TTT-3'. The amplified cDNA fragments were inserted into the BglII and HindIII restriction sites upstream of a luciferase gene in the pGL3-Basic vector (Promega, Madison, WI, USA). All plasmids were confirmed by automatic sequencing analysis.

### 3.6. Cell Culture, Transient Transfection, and Luciferase Assay

Cells were isolated from the *R. uyekii* hepatopancreas [57] and maintained in Leibovitz L-15 medium (Gibco-BRL, Gaithersburg, MD, USA) with 10% heat-inactivated fetal bovine serum (FBS; Gibco-BRL) and 1% (*v/v*) penicillin/streptomycin (PS; Gibco-BRL). Cells were seeded at a density of 1–105 cells/well in a 12-well culture plate and incubated with the indicated amount of E2 or EE2 for 16 h. The steroid in the cells was depleted by incubating the cells with L-15 supplemented with charcoal-stripped FBS (Sigma, St. Louis, MO, USA). HEK293T cells were maintained in Dulbecco’s modified Eagle’s medium (Gibco-BRL) with 10% heat-inactivated FBS (Gibco-BRL) and 1% (*v/v*) PS (Gibco-BRL) in a 37 °C incubator. For the luciferase assay, HEK293T cells were seeded in 24-well culture plates and transfected with Ru-Vg reporter and β-galactosidase expression vectors, along with ERα expression vectors, using Polyfect transfection reagent (Qiagen, Seoul, Korea). After 24 h of transfection, the cells were treated with the indicated amount of E2 or EE2 for 16 h and then lysed in cell culture lysis buffer (Promega, Madison, WI, USA). Luciferase activity was determined using an analytical luminometer (Wallac Victor2 plate reader, Perkin Elmer, Waltham, MA, USA) according to the manufacturer’s instructions (Promega, Madison, WI, USA). The luciferase activity was normalized for transfection efficiency against the corresponding β-galactosidase activity. All assays were performed at least in triplicate.

### 3.7. Statistical Analysis

All data are expressed as means ± SD (*n* = 3). Statistical significance was determined using the unpaired two-tailed Student’s *t*-test. *p*-Values less than 0.05 were considered to represent statistically significant differences.

## 4. Conclusions

In this study, we reported the molecular cloning and characterization of the Vg gene of the Korean rose bitterling *R. uyekii*. Ru-Vg contains lipovitellin I, lipovitellin II, and phosvitin domains, but not the vWFD domain or the *C*-terminal peptide. It is, therefore, classified as subtype VgAo1, according to the nomenclature of Finn and Kristoffersen [32]. The level of Ru-Vg transcripts was higher in the ovary than in the hepatopancreas and other tissues, consistent with other VgAo1-type genes. In addition, we demonstrated that Ru-Vg expression is regulated by E2 and EE2 in primary cultured cells using a luciferase assay system. These results suggest that Ru-Vg may be useful as a biomarker for exposure to E2 and EE2.
